# HLA-DRB3/4/5-based susceptibility profiles in nivolumab-induced type 1 diabetes and interstitial lung disease

**DOI:** 10.3389/fimmu.2026.1840622

**Published:** 2026-06-02

**Authors:** Shinpei Nishikido, Hiroyuki Mishima, Koh-ichiro Yoshiura, Noriho Sakamoto, Hiroshi Ishimoto, Shimpei Morimoto, Ichiro Horie, Satoru Akazawa, Ai Haraguchi, Hiromasa Tsujiguchi, Akinori Hara, Atsushi Tajima, Kazuyoshi Hosomichi, Hiroshi Mukae, Atsushi Kawakami, Norio Abiru

**Affiliations:** 1 Department of Endocrinology and Metabolism, Nagasaki University Hospital, Nagasaki, Japan; 2 Division of Advanced Preventive Medical Science, Nagasaki University Graduate School of Biomedical Sciences, Nagasaki, Japan; 3 Department of Human Genetics and Leading Medical Research Core Unit, Nagasaki University Graduate School of Biomedical Sciences, Nagasaki, Japan; 4 Department of Respiratory Medicine, Nagasaki University Graduate School of Biomedical Sciences, Nagasaki, Japan; 5 Department of Insured Medical Care Management, Nagasaki University Hospital, Nagasaki, Japan; 6 Clinical Research Center, Nagasaki University Hospital, Nagasaki, Japan; 7 Department of Hygiene and Public Health, Faculty of Medicine, Institute of Medical, Pharmaceutical and Health Sciences, Kanazawa University, Kanazawa, Japan; 8 Department of Bioinformatics and Genomics, Graduate School of Advanced Preventive Medical Sciences, Kanazawa University, Kanazawa, Japan; 9 Environmental Stress Research Center (eSRC), Kanazawa University, Kanazawa, Japan; 10 Laboratory of Computational Genomics, School of Life Science, Tokyo University of Pharmacy and Life Sciences, Tokyo, Japan; 11 Department of Immunology and Rheumatology, Division of Advanced Preventive Medical Science, Nagasaki University Graduate School of Biomedical Sciences, Nagasaki, Japan

**Keywords:** genome-wide association study, HLA class II, HLA-DRB3/4/5, immune checkpoint inhibitor, immune-related adverse events, interstitial lung disease, type 1 diabetes

## Abstract

Immune checkpoint inhibitors (ICIs) can induce severe immune-related adverse events (irAEs), including type 1 diabetes (ICI-T1D) and interstitial lung disease (ICI-ILD); however, predictive genetic markers have not yet been fully elucidated. Accordingly, this study aimed to identify genetic polymorphisms associated with susceptibility to nivolumab-induced ICI-T1D and ICI-ILD. To address this, whole-genome sequencing using next-generation sequencing was performed on genomic DNA obtained from patients recruited from multiple centers nationwide who developed ICI-T1D (n = 14), ICI-ILD (n = 58), or no irAEs (ICI-Control, n = 72) after initiation of nivolumab therapy, followed by a genome-wide association study (GWAS). The primary GWAS identified an association signal within the HLA region on chromosome 6 in the comparison between the ICI-T1D and ICI-Control groups, whereas no significant associations were detected in analyses involving the ICI-ILD group. This signal was accompanied by an increased rate of missing genotypes, most prominently around the HLA-DRB5 locus, suggesting underlying structural and haplotypic complexity within the HLA-DR region that cannot be fully resolved by reference genome–based short-read analysis alone, leading to a *post hoc* analysis focusing on disease-susceptibility alleles of HLA-DRB3/4/5 and their haplotypic structures in relation to HLA-DRB1, which is in strong linkage disequilibrium with these loci, with comparisons to a healthy reference population (General Controls; n = 1,320). 24 distinct HLA-DRB1 alleles and 11 distinct HLA-DRB3/4/5 alleles (including null types), and 30 distinct haplotypes were identified. Compared with General Controls, in the ICI-T1D group, HLA-DRB1*04:05:01, DRB1*08:02:01, and DRB4*01:03:01 were associated with susceptibility; in addition, the haplotypes DRB1*04:05:01–DRB4*01:03:01, DRB1*08:02:01–DRB3/4/5 null, and DRB1*09:01:02–DRB4*01:03:01 were also associated with susceptibility. For the ICI-ILD group, HLA-DRB1*08:03:02 was associated with susceptibility, and the haplotypes DRB1*04:10:03–DRB4*01:03:01 and DRB1*08:03:02–DRB3/4/5 null were also associated with susceptibility. Overall, these findings indicate that HLA class II polymorphisms, including DRB3/4/5, contribute to genetic susceptibility to ICI-related irAEs and support the extension of HLA typing beyond DRB1 to better elucidate the immunogenetic basis of these adverse events.

## 1 Introduction

Although immune checkpoint inhibitors (ICIs) have greatly improved outcomes in various types of cancers, they also pose new challenges in the form of immune-related adverse events (irAEs) (1). Among them, immune checkpoint inhibitor-induced type 1 diabetes (ICI-T1D) and immune checkpoint inhibitor-induced interstitial lung disease (ICI-ILD) are of particular clinical importance because they often develop acutely and can lead to severe and potentially life-threatening clinical consequences. ICI-T1D occurs rarely with an incidence of approximately 0.2%–0.9% (2, 3); however, it is associated with a substantial medical and social burden relative to its frequency, as it often presents as a severe (grade 3/4) adverse event, with diabetic ketoacidosis (DKA) reported in approximately 70% of cases (4), and irreversibly leads to insulin dependence (3). Meanwhile, ICI-ILD of Grade 3 or higher has been reported to occur in approximately 1–3% of patients in clinical trials, particularly in those with non-small cell lung cancer (5–7). Importantly, ICI-ILD is recognized as one of the most serious immune-related adverse events and a leading cause of irAE-related death (8–10). From the perspective of clinical severity, potential fatality, and the need for early detection and intervention, the establishment of disease-prediction biomarkers and the development of personalized medicine based on such biomarkers are urgent issues.

Previous studies have suggested the involvement of common HLA class II genes as genetic factors associated with disease susceptibility in ICI-T1D and conventional type 1 diabetes (T1D). In conventional T1D, HLA class II genes, specifically the DR-DQ haplotype, are known to contribute profoundly to disease susceptibility. DRB1*03:01–DQB1*02:01 (DR3) and DRB1*04:01–DQB1*03:02 (DR4) have been identified as the major susceptible haplotypes primarily in populations of European ancestry, including cohorts from Europe and North America, while also being evaluated in smaller Asian cohorts, with DR3/DR4 heterozygotes showing the highest risk (11). Meanwhile, in Japanese patients with acute-onset type 1 diabetes, DRB1*04:05–DQB1*04:01, DRB1*08:02–DQB1*03:02, and DRB1*09:01-DQB1*03:03 have been reported as disease-susceptible haplotypes, whereas DRB1*15:01–DQB1*06:02 and DRB1*15:02–DQB1*06:01 have been reported as disease-protective haplotypes (12).

HLA analyses have shown that the frequency of ICI-T1D-susceptibility-conferring HLA class II haplotypes overlapping with conventional T1D-susceptibility-conferring haplotypes, such as DR4 and DR3, was significantly higher in cohorts of patients of predominantly European ancestry than in control subjects (3, 13). Meanwhile, in the Japanese population, DRB1*04:05–DQB1*04:01 is the major disease susceptibility haplotype for ICI-T1D as well as conventional T1D, and DPB1*05:01 is reported to be an independent ICI-T1D-susceptibility allele (14). These findings suggest that ICI-T1D may develop against a background of the DR-DQ system and other genetic autoimmune predispositions shared with conventional T1D.

In conventional interstitial lung disease (ILD), the contribution of HLA class II gene polymorphisms, particularly the DRB1 molecule, to disease susceptibility has been reported in multiple studies. In cohorts of predominantly European ancestry, the DRB1*15:01–DQB1*06:02 haplotype has been identified as a major factor of disease susceptibility for idiopathic pulmonary fibrosis and fibrosing idiopathic interstitial pneumonia, and the risk for and the severity of lung fibrosis have been shown to be higher in DR15 carriers (15). In connective tissue disease-associated ILD, Furukawa et al. demonstrated that, in Japanese patients with rheumatoid arthritis (RA), HLA-DRB1 alleles encoding the shared epitope are protective against ILD, whereas DRB1*15 and DRB1*16 alleles are associated with increased susceptibility to RA-associated ILD, indicating a distinct HLA genetic background for pulmonary involvement (16). In addition, a tryptophan substitution at position 9 in the DRB1 molecule has been reported to be strongly associated with the development of RA-ILD in hospital-based Korean RA cohorts (17). Meanwhile, evidence related to genetic factors underlying ICI-ILD remains limited. Correale et al. reported increased frequencies of HLA-B35 and DRB1*11 in patients with ICI-ILD (18); however, this study was exploratory in nature due to the small number of cases. Abed et al. also conducted an exploratory analysis of the association between HLA genotypes and irAEs, including ICI-ILD, in a European cohort, suggesting a potential genetic background for ICI treatment responsiveness and toxicity, but did not identify any established risk alleles (19). These findings suggest that a pre-existing HLA-dependent immunological background may be involved in the pathogenesis of ICI-ILD, but further research is needed to clarify its genetic architecture and to identify similarities and differences relative to the HLA risk structures known for conventional autoimmune- and connective tissue disease-associated ILD.

Herein, we performed a comprehensive search for disease-associated genomic regions in ICI-T1D and ICI-ILD patients using a genome-wide association study (GWAS) based on whole-genome sequencing (WGS). In GWAS comparing patients with ICI-T1D and nivolumab-treated patients without irAEs (ICI-Control), suggestive association signals were detected in the HLA region on chromosome 6, accompanied by a marked concentration of missing genotypes near the HLA-DRB5 locus. Because read alignment in the primary GWAS was restricted to the canonical chromosomes of the GRCh38 (hg38) reference genome, which includes HLA-DRB5 but does not contain reference sequences for HLA-DRB3 and HLA-DRB4, reference genome–based short-read WGS alone is insufficient to fully evaluate the polymorphic and structural architecture of the HLA-DR region. Accordingly, to overcome these limitations and to elucidate disease-susceptibility alleles within the HLA-DR region, we performed a *post hoc* high-resolution HLA analysis using next-generation sequencing–based methods, focusing on the presence or absence and allelic structures of DRB3/4/5 and their haplotypic configurations with DRB1, which is in strong linkage disequilibrium with these loci.

## 2 Materials and methods

### 2.1 Study design and population

This study was designed as a case-control, non-interventional study in patients treated with nivolumab between September 2014 and March 2020, and was conducted as a nationwide multicenter study involving nine departments of Nagasaki University Hospital and 33 departments across 29 other institutions (**Supplementary Table 1**) between December 2017 and March 2022. A total of 144 patients were included in this study; patients who developed ICI-T1D (n = 14) or ICI-ILD (n = 58) were identified, and patients without irAEs served as controls (ICI-Control group; n = 72).

In the primary study (WGS and GWAS), genomic DNA extracted from peripheral blood samples of the ICI-T1D, ICI-ILD, and ICI-Control groups was analyzed.

*Post hoc* high-resolution HLA analysis was performed using preserved DNA obtained in the primary study. The final numbers of cases available for HLA analysis were as follows: ICI-T1D (n = 13), ICI-ILD (n = 57), and ICI-Control (n = 72). One patient who concurrently developed ICI-T1D and ICI-ILD was included in both groups.

For the *post hoc* HLA analysis, an external population-based control cohort was included. Accordingly, HLA data from a population-based cohort of residents of Shika-machi, Ishikawa Prefecture, provided by Kanazawa University, were used as controls (General Control group, n = 1,320).

### 2.2 Clinical assessments

Clinical data collected included sex, age, body height, primary disease, date of initiation and cumulative number of doses of nivolumab, body weight at initiation, and a history of prior treatment with other ICIs (anti–CTLA-4, anti–PD-1, and anti–PD-L1 antibodies).

Additional clinical information was collected according to the type of immune-related adverse event.

For the ICI-T1D group, the collected data included the date of onset of hyperglycemia-related symptoms, the date of diagnosis, body weight at the initiation of nivolumab treatment and immediately before diagnosis, as well as laboratory findings such as blood glucose, HbA1c, ketone bodies, blood gas analysis, pancreatic enzyme levels, anti-GAD antibody status, and serum and urinary C-peptide levels.

For the ICI-ILD group, the collected data included the date of onset of respiratory symptoms or detection of abnormal lung sounds (rales), the date of ILD diagnosis, details of treatment such as steroid administration and oxygen therapy, chest CT imaging findings, and biomarkers of inflammation, liver function, and lung injury (CRP, AST, ALT, γ-GTP, KL-6, SP-A, and SP-D), as well as blood gas analysis and bacteriological and cytological examination of sputum samples.

### 2.3 Primary genetic analysis: whole-genome sequencing and genome-wide association study

As the primary study, WGS and GWAS were performed using peripheral blood DNA samples obtained from the ICI-T1D, ICI-ILD, and ICI-Control groups. Library preparation was performed using the TruSeq PCR-free kit (Illumina) and 151-bp × 151-bp paired-end sequencing was carried out on the HiSeq X platform. The obtained reads were aligned to canonical chromosomes of the hg38 reference genome by Clara Parabricks version 3.7.0 to generate sample-specific gVCFs. The gVCFs were jointly genotyped using GLnexus version 1.4.1, and GWAS was performed using the “–glm firth-fallback” option in PLINK version 2 (June 21, 2023 version).

The analysis included biallelic single-nucleotide variants (SNVs) that were genotyped in more than 70% of samples, excluding variants with a minor allele frequency of <1% and those deviating from Hardy–Weinberg equilibrium (p < 1 × 10^-8^). After quality control, a total of 9,207,910 SNVs were included in the analysis.

### 2.4 *Post hoc* HLA analysis: high-resolution HLA genotyping

In the primary WGS-based GWAS of this study, read alignment was restricted to the canonical chromosomes of the hg38 reference genome. Within this canonical reference, HLA-DRB5 is represented, whereas HLA-DRB3 and HLA-DRB4 are not included, reflecting the fact that these loci are not uniformly present across all HLA-DR haplotypes. Accordingly, evaluation of the polymorphic and structural architecture of the HLA-DR region—including DRB3, DRB4, and DRB5—and its association with disease susceptibility cannot be adequately achieved using reference genome–based short-read WGS analysis alone. Based on the primary GWAS results highlighting the HLA-DR region, a *post hoc* analysis was therefore performed using high-resolution HLA typing independent of the reference genome, focusing on HLA-DRB1 and DRB3/4/5, including allele-level and haplotype-level association analyses.

For the *post hoc* analysis, high-resolution HLA genotyping of the ICI-T1D, ICI-ILD, and ICI-Control groups was performed using a sequence-capture-based next-generation sequencing (NGS) approach (20). Libraries targeting HLA gene regions were prepared and sequenced by paired-end sequencing on the Illumina NextSeq 2000 platform. HLA allele typing at third-field resolution was conducted using TypeStream Visual software (version 3.0.0.27232; One Lambda, Thermo Fisher Scientific) with the IPD-IMGT/HLA Database (version 3.52). In particular, the presence or absence and allelic structures of HLA-DRB3, DRB4, and DRB5, as well as DRB1–DRB3/4/5 haplotypes in strong linkage disequilibrium, were determined.

As an external control for the HLA analysis, HLA data from a population-based cohort of residents of Shika-machi, Ishikawa Prefecture, Japan (n = 1,320) were used as the General Control group, as previously described (21), to evaluate differences in allele and haplotype frequencies between the irAE and control groups. This cohort was used exclusively for comparative analyses of HLA allele and haplotype frequencies and was treated independently of the primary GWAS.

### 2.5 Statistical analysis

In the primary GWAS, the ICI-T1D and ICI-ILD groups were each independently compared with the ICI-Control group. A genome-wide significance threshold of p < 5.0 × 10^-8^ was used as the reference standard.

In the *post hoc* high-resolution HLA analysis, HLA allele and haplotype frequencies in the ICI-T1D and ICI-ILD groups were compared with those in the ICI-Control and General Control groups, respectively. Allele and haplotype counts were based on the number of individuals carrying each allele or haplotype (carrier-based analysis), irrespective of zygosity. This approach assumes a dominant model, which is biologically appropriate for HLA class II–restricted antigen presentation, and is widely applied in HLA association studies. Conventional allele frequencies are additionally provided in **Supplementary Tables 2A, B**. Fisher’s exact test was used for frequency comparisons between groups, and statistical analysis was performed with GraphPad Prism version 10.0.

The level of statistical significance was set at p < 0.05, and alleles or haplotypes with significantly higher or lower frequencies in the ICI-T1D and ICI-ILD groups than in the control groups were defined as disease-susceptible (susceptible) and disease-protective (protective), respectively. Moreover, p values were reported without adjustment for multiple testing, because this analysis was exploratory in nature and aimed at identifying candidate HLA alleles and haplotypes for future validation studies.

### 2.6 Ethical approval and study registration

This study was conducted in accordance with the Declaration of Helsinki and was approved by the Ethics Committee of Nagasaki University Hospital and the Ethics Review Committee of each participating institution (approval number: 17121803). This study was also registered in the University Hospital Medical Information Network (UMIN) clinical trial registration system (study ID: UMIN000030495).

The *post hoc* analysis (high-resolution HLA analysis) was conducted as a retrospective observational study using DNA samples obtained and stored in the primary study and was performed on an opt-out basis with approval from the Ethics Committee (approval number: 24100301).

## 3 Results

### 3.1 Clinical characteristics

The clinical characteristics are summarized in **Table 1**. The ICI-T1D group tended to have a lower proportion of patients with non–small cell lung cancer (ICI-T1D group: 35.7%, ICI-ILD group: 60.3%, ICI-Control group: 56.9%), and relatively high percentages of melanoma and gastric cancer (melanoma: 21.4%, 10.3%, 1.4%, gastric cancer: 21.4%, 1.7%, 2.8%). Regarding the prior use of other immune checkpoint inhibitors, anti–PD-1 antibodies other than nivolumab were more frequently used in the ICI-T1D group, and no patients received concomitant anti–CTLA-4 antibody therapy.

**Table 1:** Clinical characteristics.

	All (n = 144)	ICI-T1D (n = 14)	ICI-ILD (n = 58)	ICI-Control (n = 72)
Age, years				
Mean (SD)	66.2 (10.0)	67 (9.8)	66.1 (9.6)	66 (10.5)
Median	67	69.5	67	66
Range	43-86	44-76	43-86	43-86
Sex				
Female, n (%)	27 (18.8)	3 (21.4)	9 (15.5)	15 (20.8)
Height, cm				
Mean (SD)	164.7 (8.5)	163.1 (10.1)	165.1 (8.5)	164.8 (8.1)
Weight, kg				
Mean (SD)	60.5 (13.4)	62.4 (15.9)	60.4 (12.3)	60.2 (13.9)
Cancer type, n (%)				
NSCLC	81 (56.3)	5 (35.7)	35 (60.3)	41 (56.9)
Cervical cancer	21 (14.6)	1 (7.1)	7 (12.1)	13 (18.1)
RCC	19 (13.2)	1 (7.1)	7 (12.1)	11 (15.3)
Melanoma	10 (6.9)	3 (21.4)	6 (10.3)	1 (1.4)
Gastric cancer	6 (4.2)	3 (21.4)	1 (1.7)	2 (2.8)
Mesothelioma	6 (4.2)	1 (7.1)	1 (1.7)	4 (5.6)
Other	1 (0.7)	0 (0.0)	1 (1.7)	0 (0.0)
Prior exposure to other ICIs, n (%)*				
Overall	12 (8.3)	3 (21.4)	7 (12.1)	2 (2.8)
Anti-CTLA-4 antibody	6 (4.2)	0 (0.0)	4 (6.9)	2 (2.8)
Anti-PD-1 antibody	4 (2.8)	3 (21.4)	1 (1.7)	0 (0.0)
Anti-PD-L1 antibody	3 (2.1)	0 (0.0)	3 (5.2)	0 (0.0)
Severe disease at diagnosis, n (%)				
Cases		8 (57.1)	12 (20.7)	
Time to onset, days				
Mean (SD)		356.9 (329.5)	200.5 (221.9)	
Median		218.5	105.0	
Range		89-1036	6-845	
Clinical features of ICI-T1D				
Plasma glucose, mg/dL		641.1 (308.3)		
HbA1c, %		8.3 (1.8)		
Serum CPR (fasting), ng/mL		0.41 (0.34)		
ΔCPR (glucagon stimulation test)		0.45 (0.74)		
Urinary CPR, µg/day		10.1 (10.4)		
Anti-GAD antibody positive, n (%)		4 (28.6)		
Clinical features of ICI-ILD				
SpO_2_, %			96.2 (3.0)	
KL-6, U/mL			641.0 (746.6)	
SP-A, ng/mL			82.2 (105.1)	
SP-D, ng/mL			149.4 (93.5)	
CRP, mg/dL			3.2 (4.3)	
Treatment for ILD, n (%)				
Steroid pulse therapy			10 (17.2)	
Steroid therapy (non-pulse)			32 (55.2)	
Immunosuppressive therapy			0 (0.0)	
Other			18 (31.0)	
Chest CT findings (n = 51), n (%)^†^				
DAD			2 (3.9)	
OP			44 (86.3)	
Others (NSIP, HP)			5 (9.8)	

Data are presented as mean (SD) or number (%). SD, standard deviation; ICI, immune checkpoint inhibitor; ICI-T1D, immune checkpoint inhibitor–induced type 1 diabetes; ICI-ILD, immune checkpoint inhibitor–induced interstitial lung disease; ICI-Control, immune checkpoint inhibitor–treated patients without immune-related adverse events; NSCLC, non–small cell lung cancer; RCC, renal cell carcinoma; CTLA-4, cytotoxic T-lymphocyte–associated protein 4; PD-1, programmed cell death 1; PD-L1, programmed death-ligand 1; DAD, diffuse alveolar damage; OP, organizing pneumonia; NSIP, nonspecific interstitial pneumonia; HP, hypersensitivity pneumonitis; CT, computed tomography; CPR, C-peptide; GAD, glutamic acid decarboxylase; SpO_2_, peripheral oxygen saturation; KL-6, Krebs von den Lungen-6; SP-A, surfactant protein A; SP-D, surfactant protein D; CRP, C-reactive protein. *Some patients received more than one prior ICI therapy. ^†^Chest CT findings were available for 51 of 58 patients with ICI-ILD.

The proportion of cases classified as severe at diagnosis was 57.1% in the ICI-T1D group and 20.7% in the ICI-ILD group.

The mean time from the initiation of nivolumab administration to the onset of ICI-related adverse events (time to onset) was 356.9 ± 329.5 days (mean ± SD) in the ICI-T1D group and 200.5 ± 221.9 days (mean ± SD) in the ICI-ILD group, with a longer time to onset observed in the ICI-T1D group. Laboratory findings at the onset showed a mean blood glucose level of 641.1 mg/dL, HbA1c of 8.3%, and fasting serum CPR of 0.41 ng/mL, which, as previously reported, indicated an acute-onset clinical phenotype resembling fulminant type 1 diabetes.

In contrast, in the ICI-ILD group, 80.6% of patients presented with respiratory symptoms (e.g., dyspnea, cough), and more than 70% of patients were treated with steroids. On chest CT scans, organizing pneumonia was the most common pattern (44/51, 86.3%), whereas diffuse alveolar damage (DAD) and other patterns including nonspecific interstitial pneumonia (NSIP) and hypersensitivity pneumonitis (HP) were less frequently observed.

### 3.2 Genome-wide association analysis

**Figure 1:**
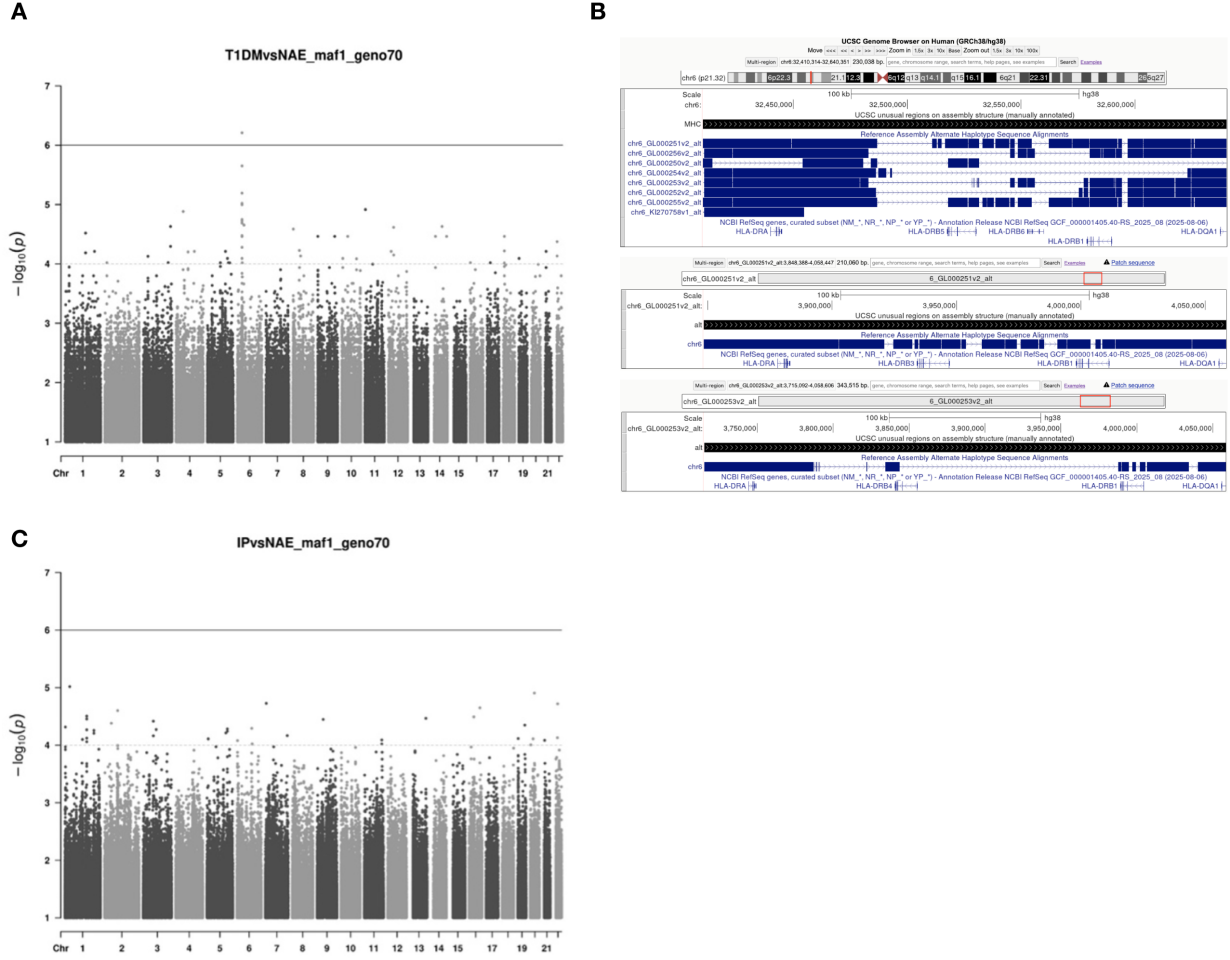
Genome-wide association study (GWAS) results comparing patients with immune checkpoint inhibitor–induced type 1 diabetes (ICI-T1D), immune checkpoint inhibitor–induced interstitial lung disease (ICI-ILD), and immune checkpoint inhibitor–treated controls without immune-related adverse events (ICI-Control). **(A)** Manhattan plot of the GWAS comparing the ICI-T1D group with the ICI-Control group using whole-genome sequencing data. A suggestive association signal was detected on chromosome 6p21.32 within the HLA class II region, accompanied by a cluster of missing genotypes. **(B)** Regional association plot of the HLA class II locus (± 1 Mb) surrounding the suggestive signal identified in panel **(A)**. The association peak was located adjacent to the HLA-DRB1/DRB3/DRB4/DRB5 gene cluster, with a marked enrichment of missing genotypes near the HLA-DRB5 locus. **(C)** Manhattan plot of the GWAS comparing the ICI-ILD group with the ICI-Control group. No genome-wide significant or suggestive association signals were observed. The dashed horizontal line indicates the suggestive significance threshold.

In the primary GWAS of this study, WGS data from the ICI-T1D, ICI-ILD, and ICI-Control groups were used for genome-wide comparative analyses. A comparison between the ICI-T1D and ICI-Control groups revealed a suggestive association signal in the HLA class II region on chromosome 6p21.32 (**Figure 1A**), with a lead variant at hg38:g.32,491,352T>C (p = 6.17 × 10^-7^) (**Table 2**).

**Table 2:** Summary of genome-wide association analysis.

Position	Odds ratio	P-value	Nearest gene	Genomic context
chr6:32,490,466 C>G	1204.8	2.23E-06	HLA-DRB5	Intergenic (within MHC class II region)
chr6:32,490,467 C>T	1204.8	2.23E-06	HLA-DRB5	Intergenic (within MHC class II region)
chr6:32,490,472 A>C	853.3	6.42E-06	HLA-DRB5	Intergenic (within MHC class II region)
chr6:32,490,479 T>A	853.3	6.43E-06	HLA-DRB5	Intergenic (within MHC class II region)
chr6:32,491,352 T>C	15.9	6.17E-07	HLA-DRB5	Intergenic (within MHC class II region)
chr6:32,491,356 A>G	10.1	9.53E-06	HLA-DRB5	Intergenic (within MHC class II region)

All variants are located within the HLA class II region on chromosome 6p21.32 and are annotated as intergenic variants proximal to the HLA-DRB5 locus based on the hg38 reference genome.

The regional plot demonstrated a concentration of missing genotypes in this region (**Figure 1B**). This location was adjacent to the HLA-DRB1/DRB3/DRB4/DRB5 gene cluster, with multiple HLA class II genes localized within ±1 Mb of the signal. Results of HLA typing using HLA-HD software revealed a high frequency of “not typed” HLA-DRB5 alleles in the ICI-T1D group (data not shown), which prompted a *post hoc*, high-resolution analysis of the HLA region.

In contrast, no genome-wide significant or suggestive association signals were observed in the ICI-ILD group (**Figure 1C**). The allele count data for GWAS are available in the Zenodo repository (22).

### 3.3 High-resolution HLA-DRB1 and DRB3/4/5 allele analysis

Based on the primary GWAS results, we performed a *post hoc* evaluation of the HLA-DR region and identified 24 distinct DRB1 alleles and 11 distinct DRB3/4/5 alleles, including null alleles.

Compared with the General Control group, DRB1*04:05:01 and DRB1*08:02:01 were significantly more frequent in the ICI-T1D group (**Table 3A**). In parallel analyses of the DRB3/4/5 loci, DRB4*01:03:01 was significantly more frequent in the ICI-T1D group (**Table 3B**).

**Table 3A:** HLA-DRB1 allele frequencies in ICI-T1D and general control.

	ICI-T1D (n = 13)		General control (n = 1320)		ICI-T1D vs. General control		
DRB1	n	%	n	%	P	OR	95% CI
*01:01:01	0	0.0	144	10.9	0.383	0.00	0.000 to 2.409
*04:01:01	0	0.0	22	1.7	>0.9999	0.00	0.000 to 19.36
*04:03:01	1	7.7	89	6.7	0.599	1.15	0.1063 to 7.451
*04:05:01	7	53.8	315	23.9	**0.020**	3.72	1.363 to 11.17
*04:06:01	1	7.7	65	4.9	0.485	1.61	0.1478 to 8.927
*04:10:01	0	0.0	51	3.9	>0.9999	0.00	0.000 to 7.588
*04:10:03	0	0.0	7	0.5	>0.9999	0.00	0.000 to 65.62
*07:01:01	0	0.0	7	0.5	>0.9999	0.00	0.000 to 65.62
*08:02:01	4	30.8	118	8.9	**0.025**	4.53	1.518 to 14.39
*08:03:02	2	15.4	218	16.5	>0.9999	0.92	0.2019 to 3.749
*09:01:02	4	30.8	404	30.6	>0.9999	1.01	0.3410 to 3.149
*10:01:01	0	0.0	18	1.4	>0.9999	0.00	0.000 to 19.56
*11:01:01	1	7.7	58	4.4	0.446	1.81	0.1663 to 10.11
*12:01:01	0	0.0	96	7.3	0.616	0.00	0.000 to 3.795
*12:02:01	0	0.0	54	4.1	>0.9999	0.00	0.000 to 7.128
*13:01:01	0	0.0	55	4.2	>0.9999	0.00	0.000 to 6.987
*13:02:01	2	15.4	142	10.8	0.643	1.51	0.3304 to 6.197
*14:03:01	0	0.0	38	2.9	>0.9999	0.00	0.000 to 10.48
*14:05:01	0	0.0	42	3.2	>0.9999	0.00	0.000 to 9.385
*14:06:01	0	0.0	37	2.8	>0.9999	0.00	0.000 to 10.79
*14:54:01	2	15.4	58	4.4	0.113	3.96	0.8563 to 16.78
*15:01:01	0	0.0	142	10.8	0.383	0.00	0.000 to 2.448
*15:02:01	0	0.0	290	22.0	0.084	0.00	0.000 to 1.039
*16:02:01	0	0.0	19	1.4	>0.9999	0.00	0.000 to 18.38

Data are presented as number of individuls carrying each allele (carrier-based analysis) or number (%). Allele counts are based on the presence of the allele irrespective of zygosity. Comparisons between groups were performed using Fisher’s exact test. P values < 0.05 are shown in bold.

**Table 3B:** HLA-DRB3/4/5 allele frequencies in ICI-T1D and general control.

	ICI-T1D (n = 13)		General control (n = 1320)		ICI-T1D vs. General control		
DRB3/4/5	n	%	n	%	P	OR	95% CI
DRB3*01:01:02	0	0.0	176	13.3	0.238	0.00	0.000 to 1.912
DRB3*02:02:01	3	23.1	207	15.7	0.443	1.61	0.4721 to 5.476
DRB3*03:01:01	2	15.4	143	10.8	0.644	1.50	0.3278 to 6.148
DRB3*03:01:03	0	0.0	46	3.5	>0.9999	0.00	0.000 to 8.494
DRB4*01:02	0	0.0	24	1.8	>0.9999	0.00	0.000 to 17.52
DRB4*01:03:01	10	76.9	473	35.8	**0.003**	5.97	1.769 to 20.30
DRB4*01:03:02	2	15.4	428	32.4	0.244	0.38	0.08345 to 1.536
DRB5*01:01:01	0	0.0	143	10.8	0.383	0.00	0.000 to 2.428
DRB5*01:02:01	0	0.0	287	21.7	0.083	0.00	0.000 to 1.053
DRB5*02:02:01	0	0.0	19	1.4	>0.9999	0.00	0.000 to 18.38
null	6	46.2	469	35.5	0.562	1.56	0.5190 to 4.239

Data are presented as number of individuals carrying each allele (carrier-based analysis) or number (%). Allele counts are based on the presence of the allele irrespective of zygosity. Comparisons between groups were performed using Fisher’s exact test. P values < 0.05 are shown in bold.

In contrast, DRB1*08:03:02 was significantly more frequent in the ICI-ILD group than in the General Control group (**Table 3C**), whereas no significant differences were observed in DRB3/4/5 allele frequencies (**Table 3D**). Notably, DRB1*04:10:03 showed a trend toward increased frequency in the ICI-ILD group (p = 0.0503).

**Table 3C:** HLA-DRB1 allele frequencies in ICI-ILD and general control.

	ICI-ILD (n = 57)		General control (n = 1320)		ICI-ILD vs. General control		
DRB1	n	%	n	%	P	OR	95% CI
*01:01:01	4	7.0	144	10.9	0.511	0.62	0.2349 to 1.581
*04:01:01	0	0.0	22	1.7	>0.9999	0.00	0.000 to 3.682
*04:03:01	2	3.5	89	6.7	0.581	0.50	0.1186 to 1.849
*04:05:01	13	22.8	315	23.9	>0.9999	0.94	0.5037 to 1.769
*04:06:01	3	5.3	65	4.9	0.758	1.07	0.3424 to 3.365
*04:10:01	4	7.0	51	3.9	0.283	1.88	0.7021 to 5.126
*04:10:03	2	3.5	7	0.5	0.050	6.82	1.400 to 31.61
*07:01:01	1	1.8	7	0.5	0.288	3.35	0.2923 to 20.03
*08:02:01	3	5.3	118	8.9	0.474	0.57	0.1823 to 1.717
*08:03:02	17	29.8	218	16.5	**0.018**	2.15	1.210 to 3.812
*09:01:02	14	24.6	404	30.6	0.379	0.74	0.4052 to 1.352
*10:01:01	0	0.0	18	1.4	>0.9999	0.00	0.000 to 4.657
*11:01:01	2	3.5	58	4.4	>0.9999	0.79	0.1853 to 2.985
*12:01:01	1	1.8	96	7.3	0.179	0.23	0.02223 to 1.255
*12:02:01	2	3.5	54	4.1	>0.9999	0.85	0.1993 to 3.235
*13:01:01	1	1.8	55	4.2	0.726	0.41	0.03983 to 2.331
*13:02:01	6	10.5	142	10.8	>0.9999	0.98	0.4440 to 2.295
*14:03:01	1	1.8	38	2.9	>0.9999	0.60	0.05800 to 3.530
*14:05:01	3	5.3	42	3.2	0.429	1.69	0.5336 to 5.118
*14:06:01	2	3.5	37	2.8	0.674	1.26	0.2920 to 4.976
*14:54:01	5	8.8	58	4.4	0.180	2.09	0.8714 to 5.376
*15:01:01	7	12.3	142	10.8	0.664	1.16	0.5041 to 2.551
*15:02:01	13	22.8	290	22.0	0.871	1.05	0.5599 to 1.973
*16:02:01	1	1.8	19	1.4	0.573	1.22	0.1150 to 7.061

Data are presented as number of individuals carrying each allele (carrier-based analysis) or number (%). Allele counts are based on the presence of the allele irrespective of zygosity. Comparisons between groups were performed using Fisher’s exact test. P values < 0.05 are shown in bold.

**Table 3D:** HLA-DRB3/4/5 allele frequencies in ICI-ILD and general control.

	ICI-ILD (n = 57)		General control (n = 1320)		ICI-ILD vs. General control		
DRB3/4/5	n	%	n	%	P	OR	95% CI
DRB3*01:01:02	3	5.3	176	13.3	0.104	0.36	0.1168 to 1.082
DRB3*02:02:01	11	19.3	207	15.7	0.459	1.29	0.6649 to 2.463
DRB3*03:01:01	6	10.5	143	10.8	>0.9999	0.97	0.4406 to 2.277
DRB3*03:01:03	2	3.5	46	3.5	>0.9999	1.01	0.2346 to 3.878
DRB4*01:02	0	0.0	24	1.8	0.621	0.00	0.000 to 3.332
DRB4*01:03:01	24	42.1	473	35.8	0.329	1.30	0.7743 to 2.185
DRB4*01:03:02	12	21.1	428	32.4	0.082	0.56	0.2799 to 1.063
DRB5*01:01:01	7	12.3	143	10.8	0.666	1.15	0.5003 to 2.530
DRB5*01:02:01	13	22.8	287	21.7	0.870	1.06	0.5673 to 1.999
DRB5*02:02:01	1	1.8	19	1.4	0.573	1.22	0.1150 to 7.061
null	23	40.4	469	35.5	0.482	1.23	0.7207 to 2.063

Data are presented as number of individuals carrying each allele (carrier-based analysis) or number (%). Allele counts are based on the presence of the allele irrespective of zygosity. Comparisons between groups were performed using Fisher’s exact test. P values < 0.05 are shown in bold.

When compared with the ICI-Control group, DRB1*08:02:01 and DRB4*01:03:01 were consistently enriched in the ICI-T1D group, whereas DRB1*15:02:01 and DRB5*01:02:01 were significantly less frequent only when compared with the ICI-Control group (**Supplementary Tables 3A, B**).

### 3.4 HLA-DRB1–DRB3/4/5 haplotype associations in ICI-T1D and ICI-ILD

Haplotype analysis was conducted using combinations of HLA-DRB1 and DRB3/4/5 alleles. In total, 30 distinct DRB1–DRB3/4/5 haplotypes were identified, and the frequency of each haplotype was compared with that in the General Control group.

In the ICI-T1D group, DRB1*04:05:01–DRB4*01:03:01, DRB1*08:02:01–DRB3/4/5 null, and DRB1*09:01:02–DRB4*01:03:01 were significantly more frequent than in the General Control group (**Table 4A**).

**Table 4A:** Haplotype frequencies in ICI-T1D and general control.

Haplotype		ICI-T1D (n = 13)		General control (n = 1320)		ICI-T1D vs. General control		
DRB1	DRB3/4/5	n	%	n	%	P	OR	95% CI
DRB1*01:01:01	null	0	0.0	144	10.9	0.383	0.00	0.000 to 2.409
DRB1*04:01:01	DRB4*01:02	0	0.0	22	1.7	>0.9999	0.00	0.000 to 19.36
DRB1*04:03:01	DRB4*01:03:01	1	7.7	72	5.5	0.521	1.44	0.1329 to 9.416
DRB1*04:05:01	DRB4*01:03:01	7	53.8	278	21.1	**0.010**	4.37	1.601 to 13.13
DRB1*04:05:01	DRB4*01:03:02	0	0.0	37	2.8	>0.9999	0.00	0.000 to 10.79
DRB1*04:06:01	DRB4*01:03:01	1	7.7	51	3.9	0.405	2.07	0.1898 to 11.63
DRB1*04:06:01	DRB4*01:03:02	0	0.0	14	1.1	>0.9999	0.00	0.000 to 26.24
DRB1*04:10:01	DRB4*01:03:01	0	0.0	40	3.0	>0.9999	0.00	0.000 to 9.902
DRB1*04:10:03	DRB4*01:03:01	0	0.0	3	0.2	>0.9999	0.00	0.000 to 122.2
DRB1*07:01:01	DRB4*01:03:01	0	0.0	4	0.3	>0.9999	0.00	0.000 to 115.2
DRB1*08:02:01	null	4	30.8	118	8.9	**0.025**	4.53	1.518 to 14.39
DRB1*08:03:02	null	2	15.4	217	16.4	>0.9999	0.92	0.2030 to 3.770
DRB1*09:01:02	DRB4*01:03:01	3	23.1	55	4.2	**0.016**	6.90	1.983 to 24.42
DRB1*09:01:02	DRB4*01:03:02	2	15.4	352	26.7	0.532	0.50	0.1100 to 2.029
DRB1*10:01:01	null	0	0.0	18	1.4	>0.9999	0.00	0.000 to 19.56
DRB1*11:01:01	DRB3*02:02:01	1	7.7	54	4.1	0.423	1.95	0.1790 to 10.93
DRB1*12:01:01	DRB3*01:01:02	0	0.0	83	6.3	>0.9999	0.00	0.000 to 4.455
DRB1*12:02:01	DRB3*03:01:01	0	0.0	2	0.2	>0.9999	0.00	0.000 to 225.5
DRB1*12:02:01	DRB3*03:01:03	0	0.0	44	3.3	>0.9999	0.00	0.000 to 8.918
DRB1*13:01:01	DRB3*01:01:02	0	0.0	55	4.2	>0.9999	0.00	0.000 to 6.987
DRB1*13:02:01	DRB3*03:01:01	2	15.4	139	10.5	0.639	1.54	0.3383 to 6.350
DRB1*13:02:01	DRB3*03:01:03	0	0.0	2	0.2	>0.9999	0.00	0.000 to 225.5
DRB1*14:03:01	DRB3*01:01:02	0	0.0	37	2.8	>0.9999	0.00	0.000 to 10.79
DRB1*14:05:01	DRB3*02:02:01	0	0.0	39	3.0	>0.9999	0.00	0.000 to 10.18
DRB1*14:06:01	DRB3*02:02:01	0	0.0	37	2.8	>0.9999	0.00	0.000 to 10.79
DRB1*14:54:01	DRB3*02:02:01	2	15.4	56	4.2	0.107	4.10	0.8877 to 17.45
DRB1*15:01:01	DRB5*01:01:01	0	0.0	142	10.8	0.383	0.00	0.000 to 2.448
DRB1*15:02:01	DRB5*01:02:01	0	0.0	286	21.7	0.083	0.00	0.000 to 1.058
DRB1*16:02:01	DRB5*02:02:01	0	0.0	19	1.4	>0.9999	0.00	0.000 to 18.38

Data are presented as number (%) of individuals carrying each haplotype (carrier-based analysis). Haplotype counts are based on the presence of each haplotype irrespective of zygosity. Comparisons between groups were performed using Fisher’s exact test. P values < 0.05 are shown in bold. A total of 30 distinct HLA-DRB1–DRB3/4/5 haplotypes were identified in this study. One haplotype observed exclusively in the ICI-Control group was not included in this table because it was absent in the ICI-T1D, ICI-ILD, and General Control groups, resulting in a 2 × 2 contingency table with all zero counts, for which Fisher’s exact test is not informative.

In the ICI-ILD group, DRB1*04:10:03–DRB4*01:03:01 and DRB1*08:03:02–DRB3/4/5 null were significantly more frequent (**Table 4B**).

**Table 4B:** Haplotype frequencies in ICI-ILD and general control.

Haplotype		ICI-ILD (n = 57)		General control (n = 1320)		ICI-ILD vs. General control		
DRB1	DRB3/4/5	n	%	n	%	P	OR	95% CI
DRB1*01:01:01	null	4	7.0	144	10.9	0.511	0.62	0.2349 to 1.581
DRB1*04:01:01	DRB4*01:02	0	0.0	22	1.7	>0.9999	0.00	0.000 to 3.682
DRB1*04:03:01	DRB4*01:03:01	2	3.5	72	5.5	0.765	0.63	0.1482 to 2.344
DRB1*04:05:01	DRB4*01:03:01	13	22.8	278	21.1	0.741	1.11	0.5905 to 2.084
DRB1*04:05:01	DRB4*01:03:02	0	0.0	37	2.8	0.400	0.00	0.000 to 2.052
DRB1*04:06:01	DRB4*01:03:01	2	3.5	51	3.9	>0.9999	0.90	0.2113 to 3.450
DRB1*04:06:01	DRB4*01:03:02	1	1.8	14	1.1	0.471	1.67	0.1542 to 10.40
DRB1*04:10:01	DRB4*01:03:01	4	7.0	40	3.0	0.105	2.42	0.8957 to 6.781
DRB1*04:10:03	DRB4*01:03:01	2	3.5	3	0.2	**0.016**	15.96	2.770 to 78.95
DRB1*07:01:01	DRB4*01:03:01	1	1.8	4	0.3	0.191	5.88	0.4722 to 36.18
DRB1*08:02:01	null	3	5.3	118	8.9	0.474	0.57	0.1823 to 1.717
DRB1*08:03:02	null	17	29.8	217	16.4	**0.017**	2.16	1.217 to 3.834
DRB1*09:01:02	DRB4*01:03:01	3	5.3	55	4.2	0.730	1.28	0.4063 to 3.771
DRB1*09:01:02	DRB4*01:03:02	11	19.3	352	26.7	0.282	0.66	0.3432 to 1.245
DRB1*10:01:01	null	0	0.0	18	1.4	>0.9999	0.00	0.000 to 4.657
DRB1*11:01:01	DRB3*02:02:01	2	3.5	54	4.1	>0.9999	0.85	0.1993 to 3.235
DRB1*12:01:01	DRB3*01:01:02	1	1.8	83	6.3	0.253	0.27	0.02595 to 1.476
DRB1*12:02:01	DRB3*03:01:01	0	0.0	2	0.2	>0.9999	0.00	0.000 to 50.35
DRB1*12:02:01	DRB3*03:01:03	2	3.5	44	3.3	0.716	1.05	0.2454 to 4.079
DRB1*13:01:01	DRB3*01:01:02	1	1.8	55	4.2	0.726	0.41	0.03983 to 2.331
DRB1*13:02:01	DRB3*03:01:01	6	10.5	139	10.5	>0.9999	1.00	0.4546 to 2.233
DRB1*13:02:01	DRB3*03:01:03	0	0.0	2	0.2	>0.9999	0.00	0.000 to 50.35
DRB1*14:03:01	DRB3*01:01:02	1	1.8	37	2.8	>0.9999	0.62	0.05958 to 3.639
DRB1*14:05:01	DRB3*02:02:01	3	5.3	39	3.0	0.250	1.82	0.5746 to 5.572
DRB1*14:06:01	DRB3*02:02:01	2	3.5	37	2.8	0.674	1.26	0.2920 to 4.976
DRB1*14:54:01	DRB3*02:02:01	5	8.8	56	4.2	0.103	2.17	0.9030 to 5.252
DRB1*15:01:01	DRB5*01:01:01	7	12.3	142	10.8	0.664	1.16	0.5041 to 2.551
DRB1*15:02:01	DRB5*01:02:01	13	22.8	286	21.7	0.870	1.07	0.5698 to 2.009
DRB1*16:02:01	DRB5*02:02:01	1	1.8	19	1.4	0.573	1.22	0.1150 to 7.061

Data are presented as number (%) of individuals carrying each haplotype (carrier-based analysis). Haplotype counts are based on the presence of each haplotype irrespective of zygosity. Comparisons between groups were performed using Fisher’s exact test. P values < 0.05 are shown in bold. A total of 30 distinct HLA-DRB1–DRB3/4/5 haplotypes were identified in this study. One haplotype observed exclusively in the ICI-Control group was not included in this table because it was absent in the ICI-T1D, ICI-ILD, and General Control groups, resulting in a 2 × 2 contingency table with all zero counts, for which Fisher’s exact test is not informative.

### 3.5 Haplotypic interactions between HLA-DRB1 and DRB3/4/5 in ICI-T1D

Haplotype frequencies of DRB1–DRB3/4/5 combinations were compared between the ICI-T1D and General Control groups. To evaluate frequency differences for each combination, a two-dimensional contingency table was constructed with DRB1 alleles as columns and DRB3/4/5 alleles as rows (**Table 5**).

**Table 5:** P values for HLA-DRB1–DRB3/4/5 haplotype frequency comparisons between ICI-T1D and general controls, with P values for each allele frequency by row (DRB1) and column (DRB3/4/5).

Allele	DRB3				DRB4			DRB5				
DRB1	*01:01:02	*02:02:01	*03:01:01	*03:01:03	*01:02	*01:03:01	*01:03:02	*01:01:01	*01:02:01	*02:02:01	null	*P*
*01:01:01											0.383	0.383
*04:01:01					>0.9999							>0.9999
*04:03:01						0.521						0.599
*04:05:01						**0.010**	>0.9999					**0.020**
*04:06:01						0.405	>0.9999					0.485
*04:10:01						>0.9999						>0.9999
*04:10:03						>0.9999						>0.9999
*07:01:01						>0.9999						>0.9999
*08:02:01											**0.025**	**0.025**
*08:03:02											>0.9999	>0.9999
*09:01:02						**0.016**	0.532					>0.9999
*10:01:01											>0.9999	>0.9999
*11:01:01		0.423										0.446
*12:01:01	>0.9999											0.616
*12:02:01			>0.9999	>0.9999								>0.9999
*13:01:01	>0.9999											>0.9999
*13:02:01			0.639	>0.9999								0.643
*14:03:01	>0.9999											>0.9999
*14:05:01		>0.9999										>0.9999
*14:06:01		>0.9999										>0.9999
*14:54:01		0.107										0.113
*15:01:01								0.383				0.383
*15:02:01									0.083			0.084
*16:02:01										>0.9999		>0.9999
***P***	0.238	0.443	0.644	>0.9999	>0.9999	**0.003**	0.244	0.383	0.083	>0.9999	0.562	

Each allele or haplotype frequency was analyzed using Fisher’s exact test. Rows represent HLA-DRB1 alleles and columns represent HLA-DRB3/4/5 alleles. Gray shading indicates haplotypes containing DRB4*01:03:01 and the associated DRB1 alleles. P, P value. P values less than 0.05 are shown in bold. A total of 30 distinct HLA-DRB1–DRB3/4/5 haplotypes were identified in this study. One haplotype observed exclusively in the ICI-Control group was not included in this table because it was absent in the ICI-T1D, ICI-ILD, and General Control groups, resulting in a 2 × 2 contingency table with all zero counts, for which Fisher’s exact test is not informative.

In the ICI-T1D group, DRB1*08:02:01 was exclusively observed in combination with DRB3/4/5 null, and the DRB1*08:02:01–DRB3/4/5 null haplotype was significantly more frequent than in the General Control group (p < 0.05).

DRB4*01:03:01 was observed in combination with multiple DRB1 alleles, of which the DRB1*04:05:01–DRB4*01:03:01 and DRB1*09:01:02–DRB4*01:03:01 combination was significantly more frequent (p < 0.05).

In the ICI-ILD group, the combination of DRB1*04:10:03 with DRB4*01:03:01 showed a more pronounced difference in frequency than the allele-level analysis alone. Although DRB1*04:10:03 alone showed only a borderline increase in frequency (odds ratio [OR] 6.82, p = 0.0503), DRB1*04:10:03–DRB4*01:03:01 was significantly more frequent than in the General Control group (OR 15.96, p = 0.016, 95% CI 2.770-78.95) (**Supplementary Table 4**).

### 3.6 HLA-DRB1–DRB3/4/5 genotype associations in ICI-T1D and ICI-ILD

In this study, we defined disease-susceptible haplotypes of ICI-T1D and ICI-ILD based on the combinations of HLA-DRB1 and DRB3/4/5 and examined the association between genotype categories and disease onset (**Tables 6A**, **B**).

**Table 6A:** DRB1-DRB3/4/5 genotypes in ICI-T1D and general control.

	ICI-T1D		General control		ICI-T1D vs. General control	
Genotype	n	%	n	%	p	OR
DRB1*04:05:01–DRB4*01:03:01/DRB1*04:05:01–DRB4*01:03:01	1	7.7	18	1.4	0.055	6.03
DRB1*04:05:01–DRB4*01:03:01/DRB1*08:02:01–DRB3/4/5 null	2	15.4	11	0.8	**<0.001**	21.64
DRB1*04:05:01–DRB4*01:03:01/DRB1*09:01:02–DRB4*01:03:01	1	7.7	12	0.9	**0.013**	9.08
DRB1*08:02:01–DRB3/4/5 null/DRB1*08:02:01–DRB3/4/5 null	0	0.0	7	0.5	0.792	0.00
DRB1*08:02:01–DRB3/4/5 null/DRB1*09:01:02–DRB4*01:03:01	0	0.0	7	0.5	0.792	0.00
DRB1*09:01:02–DRB4*01:03:01/DRB1*09:01:02–DRB4*01:03:01	0	0.0	2	0.2	0.888	0.00
DRB1*04:05:01–DRB4*01:03:01/other haplotypes	3	23.1	237	18.0	0.632	1.37
DRB1*08:02:01–DRB3/4/5 null/other haplotypes	2	15.4	93	7.0	0.245	2.40
DRB1*09:01:02–DRB4*01:03:01/other haplotypes	2	15.4	34	2.6	**0.005**	6.88

Each genotype frequency was analyzed using Fisher’s exact test. n, number of individuals with each genotype; %, frequency of the genotype; p, p value; OR, odds ratio. Susceptible haplotypes were DRB1*04:05:01–DRB4*01:03:01, DRB1*08:02:01–DRB3/4/5 null, and DRB1*09:01:02–DRB4*01:03:01. Other haplotypes refer to haplotypes other than the susceptible haplotypes listed above. P values less than 0.05 are shown in bold.

**Table 6B:** DRB1-DRB3/4/5 genotypes in ICI-ILD and general control.

	ICI-ILD		General control		ICI-ILD vs. General control	
Genotype	n	%	n	%	p	OR
DRB1*04:10:03–DRB4*01:03:01/DRB1*04:10:03–DRB4*01:03:01	0	0.0	0	0.0	n.c.	n.c.
DRB1*04:10:03–DRB4*01:03:01/DRB1*08:03:02–DRB3/4/5 null	0	0.0	0	0.0	n.c.	n.c.
DRB1*08:03:02–DRB3/4/5 null/DRB1*08:03:02–DRB3/4/5 null	1	1.8	22	1.7	0.960	1.05
DRB1*04:10:03–DRB4*01:03:01/other haplotypes	2	3.5	3	0.2	**<0.001**	15.96
DRB1*08:03:02–DRB3/4/5 null/other haplotypes	16	28.1	195	14.8	**0.006**	2.25

Each genotype frequency was analyzed using Fisher’s exact test. n, number of individuals with each genotype; %, genotype frequency; p, p value; OR, odds ratio; n.c., not calculated. Susceptible haplotypes were DRB1*04:10:03–DRB4*01:03:01 and DRB1*08:03:02–DRB3/4/5 null. Other haplotypes refer to haplotypes other than DRB1*04:10:03–DRB4*01:03:01 and DRB1*08:03:02–DRB3/4/5 null. P values less than 0.05 are shown in bold.

In ICI-T1D, the haplotypes DRB1*04:05:01–DRB4*01:03:01, DRB1*08:02:01–DRB3/4/5 null, and DRB1*09:01:02–DRB4*01:03:01 were identified as susceptible haplotypes, whereas haplotypes other than the susceptible haplotypes were grouped as other haplotypes. Genotype-based comparative analyses showed that heterozygous genotype combinations including DRB1*04:05:01–DRB4*01:03:01/DRB1*08:02:01–DRB3/4/5 null, DRB1*04:05:01–DRB4*01:03:01/DRB1*09:01:02–DRB4*01:03:01, and DRB1*09:01:02–DRB4*01:03:01/other haplotypes were significantly more frequent in the ICI-T1D group and were therefore considered disease-susceptible genotypes. These findings suggest that multiple susceptible haplotypes coexist and that heterozygous combinations of these haplotypes may further increase the risk of developing ICI-T1D.

In contrast, in ICI-ILD, the haplotypes DRB1*04:10:03–DRB4*01:03:01 and DRB1*08:03:02–DRB3/4/5 null were identified as susceptible haplotypes. In the comparison between the ICI-ILD and General Control groups, the genotype combinations DRB1*04:10:03–DRB4*01:03:01/other haplotypes and DRB1*08:03:02–DRB3/4/5 null/other haplotypes were significantly enriched in the ICI-ILD group and were therefore considered disease-susceptible genotypes. These results indicate that certain HLA genotypes may contribute to susceptibility in a disease-specific manner in the development of ICI-ILD and suggest that the genetic architecture underlying ICI-ILD differs from that observed in ICI-T1D.

## 4 Discussion

Herein, we comprehensively examined the clinical characteristics and genetic backgrounds of ICI-T1D and ICI-ILD. By integrating genome-wide association signals with high-resolution HLA analyses, this study delineates disease-specific susceptibility architectures that converge on the HLA-DR region and identifies candidate susceptibility factors.

Clinically, the ICI-T1D group tended to have a lower proportion of patients with non-small cell lung cancer and a higher proportion of patients with melanoma and gastric cancer than the ICI-ILD group, and the interval from initiation of ICI therapy to disease onset tended to be longer. The clinical features of ICI-T1D were consistent with previous reports (3, 23–25), including marked hyperglycemia at presentation, profound impairment of insulin secretion, and rapid progression to insulin-dependent diabetes.

In the primary GWAS, a comparison between the ICI-T1D and ICI-Control groups revealed suggestive association signals within the HLA class II region, particularly near the HLA-DRB5 locus. These findings suggest that the HLA-DR region may represent a key locus influencing disease susceptibility in ICI-T1D.

The interpretation of GWAS signals in the HLA region requires careful consideration due to its complex genetic architecture. The MHC region is characterized by extensive linkage disequilibrium, which complicates the identification of causal genes and alleles (26). In such regions, SNP-based GWAS signals often reflect underlying haplotypic structures rather than independent causal variants. Consistently, HLA types represent haplotypes of multiple coding and regulatory variants, and haplotype-based analyses provide greater explanatory power than single-variant approaches (27). In the present study, the suggestive GWAS signal was localized to the HLA class II region, particularly near the HLA-DRB5 locus, where multiple HLA-DRB genes are clustered. High-resolution HLA typing subsequently identified susceptibility-associated alleles and DRB1–DRB3/4/5 haplotypes within the same region. These findings indicate that our HLA analysis provides a higher-resolution interpretation of the GWAS signal rather than an independent analysis. Furthermore, the genome-wide analysis supports that no susceptibility loci outside the HLA region were identified, reinforcing the biological relevance of focusing on this locus.

The HLA-DR region is usually classified into five antigenic types (DR1, DR51, DR52, DR53, and DR8) accompanied by DRB1 plus either DRB3, DRB4, or DRB5, and each type is known to show strong linkage disequilibrium with a specific DRB1 allele. More specifically, DR51, DR52, and DR53 groups are structurally characterized by the presence of DRB5, DRB3, and DRB4, respectively, while DR1 and DR8 groups lack DRB3/4/5. These DRB3/4/5 genes are inherited in haplotype units linked to DRB1 and are thought to influence the antigen presentation properties of HLA-DR molecules and immune responses (28–30). These findings indicate the need to understand the HLA-DR region not as DRB1 alone, but as a haplotype unit including DRB3/4/5, and support the possibility that structural diversity and differential expression of DR molecules may influence the immune responses.

Detailed HLA class II analysis performed as a *post hoc* analysis showed a clear disease susceptibility associated with HLA-DRB1 in ICI-T1D. Significant disease susceptibility was observed in DRB1*04:05:01 and DRB1*08:02:01 at the allele level and in heterozygous combinations of DRB1*04:05:01–DRB4*01:03:01/DRB1*08:02:01–DRB3/4/5 null, DRB1*04:05:01–DRB4*01:03:01/DRB1*09:01:02–DRB4*01:03:01, and DRB1*09:01:02–DRB4*01:03:01/other haplotypes. A previous study from Japan (14) also reported the involvement of DRB1*04:05 in ICI-T1D, which is consistent with the present findings. In addition, our results newly suggest that DRB1*08:02:01 may represent another susceptible allele for ICI-T1D. These alleles constitute disease-susceptible DRB1–DQB1 haplotypes that have been well established in conventional T1D in the Japanese population (12). This observation indicates that the immunogenetic background of conventional T1D and ICI-T1D is at least partially overlapping, and further suggests that the susceptibility architecture of ICI-T1D may be broader and more heterogeneous than previously recognized.

Although HLA class II molecules are generally considered to be functionally defined at the two-field resolution level, we observed differences in clinical outcomes even among individuals carrying identical two-field alleles. This finding is consistent with previous reports suggesting that genetic variation beyond the two-field level may influence disease susceptibility, as higher-resolution HLA alleles can capture differences not reflected at the two-field level (31). Notably, HLA allele nomenclature distinguishes variants beyond the two-field level, where the third-field resolution captures synonymous variants that do not alter the amino acid sequence but may still affect gene expression or immune function. In addition, synonymous and regulatory variants within HLA alleles have been reported to affect gene expression and immune function, including dynamic allele-specific expression during T-cell activation (32, 33). Furthermore, HLA class II alleles may shape T-cell receptor repertoires, thereby influencing downstream immune responses (34). Taken together, these observations indicate that two-field HLA resolution alone may not fully reflect susceptibility to ICI-related adverse events.

In this study, our HLA analysis extended to the DRB3/4/5 gene region, which showed newly emerging suggestive signals in the GWAS. In the ICI-T1D group, DRB4*01:03:01 was identified as a susceptibility allele. Compared to the ICI-Control group, the frequency of DRB5*01:02:01 was significantly lower in the ICI-T1D group. DRB5*01:02:01 is known to be in strong linkage disequilibrium with DRB1*15:02:01, an allele that confers protection against T1D in the Japanese population (DR51 group). Notably, in this study, none of the 13 patients with ICI-T1D carried this allele. The absence of DRB5 in the ICI-T1D group reflects the actual lack of DRB5-positive haplotypes; however, in the primary GWAS, this difference may have been accentuated and detected as “missing” genotypes due to read mapping restricted to the canonical chromosomes of the hg38 reference genome, which includes DRB5 but not DRB3 and DRB4 as described in the Materials and Methods.

In ICI-T1D, haplotype analysis indicated that the DRB1*04:05:01-DRB4*01:03:01, DRB1*09:01:02–DRB4*01:03:01, and DRB1*08:02:01–DRB3/4/5 null haplotypes were associated with disease susceptibility. Notably, DRB4*01:03:01 was suggested to act as a modifying factor that enhanced disease susceptibility when combined with specific DRB1 alleles, particularly DRB1*04:05:01, and DRB1*09:01:02.

The HLA-DR molecule is a heterodimer composed of an invariant DRA chain and one of several polymorphic DRB chains (35, 36). Although the major polymorphism resides in DRB1, the DRB3, DRB4, and DRB5 genes are also located on the same chromosome and are in strong linkage disequilibrium with specific DRB1 alleles (37). Thus, in addition to HLA-DR molecules derived from DRB1, those encoded by DRB3/4/5 can also be expressed. This enables multiple distinct HLA-DR molecules to function in parallel within a single individual, potentially modifying the repertoire of self-antigens that can be presented as well as the geometry of T-cell receptor recognition (26). DRB3/4/5 polymorphisms should not be regarded merely as linkage markers, but rather as functional determinants that provide a molecular basis for organ-specific susceptibility to autoimmune diseases. For a long time, DRB3/4/5 were underestimated because they could not be reliably identified using conventional serological or PCR-based typing methods. The recent widespread adoption of next-generation sequencing has enabled high-resolution HLA typing, revealing associations between DRB3/4/5 and disease susceptibility in multiple autoimmune diseases such as multiple sclerosis, autoimmune hepatitis, and Sjögren’s syndrome (38–40).

In conventional T1D, DRB3*02:02 has been shown to act as a modifier of disease risk in the context of DRB1*03:01 haplotypes (41). Furthermore, a large cohort study of Swedish patients with childhood- and adolescent-onset T1D identified DRB4*01:03:01, DRB3*01:01:02, and DRB3*02:02:01 as susceptible alleles, and demonstrated that DRB4*01:01:01, DRB5*01:01, and DRB5*01:02 confer resistance (protection) (42). Moreover, a 15-residue amino acid polymorphism shared by DRB3, DRB4, and DRB5 is located within the peptide-binding groove and the CD4-binding interface, and has been suggested to directly influence antigen presentation efficiency and T-cell activation (43). These findings indicate that disease susceptibility associated with the HLA class II region may be shaped not only by the “DRB1–DQB1 axis” but also by the “DRB3/4/5 axis,” thereby providing an immunogenetic basis for defining thresholds of autoimmune responses and differences in organ specificity.

The present study suggests that DRB4*01:03:01, which has been reported to be associated with disease susceptibility in conventional T1D, is also associated with susceptibility to ICI-T1D as a single allele and that this susceptibility is further enhanced when DRB4*01:03:01 is combined with specific DRB1 alleles such as DRB1*04:05:01 and DRB1*09:01:02. These results indicate that susceptibility structures shared with conventional T1D may be embedded in the genetic architecture of the HLA-DR region, involving not only DRB1 but also DRB3/4/5, and may become evident under the unique immune environment induced by ICIs. While the present study identified associations at the haplotype level, the underlying biological mechanisms remain to be elucidated.

In ICI-ILD, DRB1*08:03:02 was associated with disease susceptibility, whereas no disease-susceptibility alleles were identified for DRB3/4/5 alone. However, haplotype-based analysis revealed significant associations for DRB1*04:10:03–DRB4*01:03:01 and DRB1*08:03:02–DRB3/4/5 null, indicating that DRB3/4/5 may contribute to susceptibility in a haplotype-dependent manner. Notably, the presence of DRB4*01:03:01 increased the odds ratio associated with DRB1*04:10:03.

In conventional ILD, disease susceptibility has been reported to be associated with specific HLA-DRB1 alleles depending on disease subtype (16, 44, 45), whereas in ICI-ILD, emerging evidence suggests involvement of distinct DRB1 alleles in the context of immune checkpoint blockade. Previous study suggested associations between ICI-ILD and specific HLA alleles, such as DRB1*11 and HLA-B*35; however, these analyses were limited and did not include comprehensive evaluation of the HLA class II region incorporating DRB3/4/5 (18). In this context, the present study is the first to demonstrate disease susceptibility associated with DRB3/4/5-containing haplotypes, such as DRB1*08:03:02–DRB3/4/5 null and DRB1*04:10:03–DRB4*01:03:01, in patients with ICI-ILD.

This study has several limitations. First, the sample size was relatively small. Because ICI-T1D is a rare irAE, the limited number of cases reduced the statistical power, particularly for sub-haplotype analyses. Accordingly, the present study should be regarded as exploratory and hypothesis-generating in nature, and the findings require validation in larger independent cohorts. In addition, given the exploratory nature of this study, the multiple allele- and haplotype-level comparisons performed, and the fact that the GWAS signals did not reach genome-wide significance, the reported associations should be interpreted with caution and considered as candidate findings requiring further validation. Second, this study was conducted in a single ethnic population (Japanese), and therefore the generalizability of the findings to other populations may be limited. Given the substantial ethnic differences in HLA allele frequencies, further studies in diverse populations are required to validate these findings. Third, owing to the observational nature of the study, a direct causal relationship between HLA polymorphisms and pathogenic mechanisms could not be established, and functional validation remains an important subject for future research. Accordingly, the identified HLA alleles and haplotypes should be interpreted as candidate susceptibility factors that warrant independent validation in larger, multi-ethnic cohorts, ideally incorporating functional studies.

As a potential clinical application, the findings of this study may contribute to risk stratification of irAEs in patients receiving ICIs. For example, comprehensive HLA typing, including DRB3/4/5-related haplotypes, could be used to proactively assess the risk of developing ICI-T1D, thereby enabling intensified glucose monitoring and earlier therapeutic intervention. Moreover, for ICI-ILD, known HLA backgrounds shared with autoimmune lung diseases may be incorporated into predictive models, which could facilitate personalized risk assessment and management. Accordingly, our findings may contribute to the more appropriate use of ICIs based on individual HLA backgrounds and support the future development of antigen-specific immunomodulatory strategies.

## Data Availability

The original raw sequencing datasets presented in this article are not readily available due to ethical and privacy restrictions to protect human participant anonymity. Requests to access the datasets should be directed to the corresponding author. The aggregated and anonymized statistical data (allele frequency count table) supporting the conclusions of this article are publicly available in Zenodo at: https://doi.org/10.5281/zenodo.19227951.
