# Supplementary material for: HLA-DRB3/4/5-based susceptibility profiles in nivolumab-induced type 1 diabetes and interstitial lung disease
Source: Front Immunol. 2026 Jun 2;17:1840622. doi: 10.3389/fimmu.2026.1840622 (PMC13269219; doi:10.3389/fimmu.2026.1840622)
Supplement: Supplementary file 1 [file Table1.docx]

Supplementary Material

## Supplementary Tables

**Supplementary Table 1.** **Participating institutions and principal investigators of primary GWAS**

| **Institution** | **Department** | **Principal investigator** |
| --- | --- | --- |
| Nagasaki University Hospital | Immunology and Rheumatology | Atsushi Kawakami^1^ |
| Nagasaki University Hospital | Respiratory Medicine | Hiroshi Mukae |
| Nagasaki University Hospital | Endocrinology and Metabolism | Norio Abiru |
| Nagasaki University Hospital | Clinical Oncology | Minoru Fukuda |
| Nagasaki University Hospital | Clinical Research Center | Hiroshi Yamamoto |
| Nagasaki University Hospital | Gastroenterology and Hepatology | Kazuhiko Nakao |
| Nagasaki University Hospital | Urology and Renal Transplantation | Hideki Sakai |
| Nagasaki University Hospital | Dermatology and Allergology | Hiroyuki Murota |
| Nagasaki University Hospital | Otolaryngology-Head and Neck Surgery | Kenichi Kaneko |
| National Cancer Center Hospital | Thoracic Oncology | Yutaka Fujiwara |
| National Cancer Center Hospital | Dermatologic Oncology | Naoya Yamazaki |
| Tochigi Cancer Center | Thoracic Oncology | Yoichi Nakamura |
| Kyushu University Hospital | Respiratory Medicine | Yoichi Nakanishi |
| Kurume University School of Medicine | Division of Respirology, Neurology, and Rheumatology, Department of Medicine, | Tomoaki Hoshino |
| University of Occupational and Environmental Health | Respiratory Medicine | Kazuhiro Yatera |
| Fukuoka University | Respiratory Medicine, Faculty of Medicine, | Masaki Fujita |
| Fukuoka University | School of Medicine, Endocrinology and Diabetes Mellitus | Takashi Nomiyama |
| Oita University | Respiratory Medicine and Infectious Diseases | Jun-ichi Kadota |
| Kumamoto University Hospital | Respiratory Medicine | Takuro Sakagami |
| Kagoshima University | Pulmonary Medicine | Hiromasa Inoue |
| Kagoshima University | Diabetes and Endocrine Medicine | Yoshihiko Nishio |
| University of the Ryukyus | Infectious, Respiratory and Digestive Medicine | Jiro Fujita |
| University of the Ryukyus | Division of Endocrinology and Metabolism, Second Department of Internal Medicine | Hiroaki Masuzaki |
| University of Miyazaki | Division of Neurology, Respirology, Endocrinology and Metabolism, Department of Internal Medicine, | Hiroaki Ueno |
| Saga University | Division of Hematology, Respiratory Medicine and Oncology, Department of Internal Medicine | Naoko Aragane |
| Tokai University School of Medicine | Division of Nephrology, Endocrinology and Metabolism, Department of Internal Medicine | Yoshitaka Mori |
| International Medical Center, Saitama Medical University | Respiratory Medicine | Hiroshi Kagamu |
| Hokkaido University | Rheumatology, Endocrinology and Nephrology | Akinobu Nakamura |
| Kanazawa University Hospital | Respiratory Medicine | Seiji Yano |
| Kyoto Prefectural University of Medicine | Pulmonary Medicine | Koichi Takayama |
| Okayama University | Nephrology, Rheumatology, Endocrinology and Metabolism | Jun Wada |
| Chiba University Graduate School of Medicine | Respirology | Shunichiro Iwasawa |
| Japanese Red Cross Nagasaki Genbaku Hospital | Respiratory Medicine | Masaaki Fuuda |
| National Hospital Organization Nagasaki Medical Center | Respiratory Medicine | Seiji Nagashima |
| JCHO Isahaya General Hospital | Respiratory Medicine | Seiji Doi |
| Nagasaki Prefecture Shimabara Hospital | Respiratory Medicine | Akitoshi Kinoshita |
| Sasebo City General Hospital | Respiratory Medicine | Hiromi Tomono |
| Kitakyusyu Munincipal Medical Center | Respiratory Medicine | Koji Inoue |
| National Hospital Organization Fukuoka Higashi Medical Center | Respiratory Medicine | Keiichi Ota |
| National Hospital Organization Ureshino Medical Center | Respiratory Medicine | Katsumi Nakatomi |
| JCHO-Kyusyu Hospital | Respiratory Medicine | Taishi Harada |
| Kagoshima City Hospital | Respiratory Medicine | Ikkou Higashimoto |

^1^Principal investigator of the primary GWAS

**Supplementary Table 2a. Carrier-based counts and allele counts of HLA-DRB1 alleles in each group**

|  | ***ICI-T1D (n=13)*** | | | | ***ICI-ILD (n=57)*** | | | | ***General Control (n=1320)*** | | | |
| --- | --- | --- | --- | --- | --- | --- | --- | --- | --- | --- | --- | --- |
|  | ***Carrier-based counts*** | | ***Allele counts*** | | ***Carrier-based counts*** | | ***Allele counts*** | | ***Carrier-based counts*** | | ***Allele counts*** | |
| ***DRB1*** | ***n*** | ***%*** | ***n*** | ***%*** | ***n*** | ***%*** | ***n*** | ***%*** | ***n*** | ***%*** | ***n*** | ***%*** |
| *01:01:01 | 0 | 0.0 | 0 | 0.0 | 4 | 7.0 | 5 | 4.4 | 144 | 10.9 | 150 | 5.7 |
| *04:01:01 | 0 | 0.0 | 0 | 0.0 | 0 | 0.0 | 0 | 0.0 | 22 | 1.7 | 22 | 0.8 |
| *04:03:01 | 1 | 7.7 | 1 | 3.8 | 2 | 3.5 | 2 | 1.8 | 89 | 6.7 | 90 | 3.4 |
| *04:05:01 | 7 | 53.8 | 8 | 30.8 | 13 | 22.8 | 15 | 13.2 | 315 | 23.9 | 336 | 12.7 |
| *04:06:01 | 1 | 7.7 | 1 | 3.8 | 3 | 5.3 | 3 | 2.6 | 65 | 4.9 | 68 | 2.6 |
| *04:10:01 | 0 | 0.0 | 0 | 0.0 | 4 | 7.0 | 4 | 3.5 | 51 | 3.9 | 52 | 2.0 |
| *04:10:03 | 0 | 0.0 | 0 | 0.0 | 2 | 3.5 | 2 | 1.8 | 7 | 0.5 | 7 | 0.3 |
| *07:01:01 | 0 | 0.0 | 0 | 0.0 | 1 | 1.8 | 1 | 0.9 | 7 | 0.5 | 7 | 0.3 |
| *08:02:01 | 4 | 30.8 | 4 | 15.4 | 3 | 5.3 | 3 | 2.6 | 118 | 8.9 | 125 | 4.7 |
| *08:03:02 | 2 | 15.4 | 2 | 7.7 | 17 | 29.8 | 18 | 15.8 | 218 | 16.5 | 240 | 9.1 |
| *09:01:02 | 4 | 30.8 | 5 | 19.2 | 14 | 24.6 | 14 | 12.3 | 404 | 30.6 | 431 | 16.3 |
| *10:01:01 | 0 | 0.0 | 0 | 0.0 | 0 | 0.0 | 0 | 0.0 | 18 | 1.4 | 18 | 0.7 |
| *11:01:01 | 1 | 7.7 | 1 | 3.8 | 2 | 3.5 | 3 | 2.6 | 58 | 4.4 | 61 | 2.3 |
| *12:01:01 | 0 | 0.0 | 0 | 0.0 | 1 | 1.8 | 1 | 0.9 | 96 | 7.3 | 97 | 3.7 |
| *12:02:01 | 0 | 0.0 | 0 | 0.0 | 2 | 3.5 | 2 | 1.8 | 54 | 4.1 | 56 | 2.1 |
| *13:01:01 | 0 | 0.0 | 0 | 0.0 | 1 | 1.8 | 1 | 0.9 | 55 | 4.2 | 56 | 2.1 |
| *13:02:01 | 2 | 15.4 | 2 | 7.7 | 6 | 10.5 | 7 | 6.1 | 142 | 10.8 | 147 | 5.6 |
| *14:03:01 | 0 | 0.0 | 0 | 0.0 | 1 | 1.8 | 1 | 0.9 | 38 | 2.9 | 39 | 1.5 |
| *14:05:01 | 0 | 0.0 | 0 | 0.0 | 3 | 5.3 | 3 | 2.6 | 42 | 3.2 | 43 | 1.6 |
| *14:06:01 | 0 | 0.0 | 0 | 0.0 | 2 | 3.5 | 2 | 1.8 | 37 | 2.8 | 39 | 1.5 |
| *14:54:01 | 2 | 15.4 | 2 | 7.7 | 5 | 8.8 | 5 | 4.4 | 58 | 4.4 | 60 | 2.3 |
| *15:01:01 | 0 | 0.0 | 0 | 0.0 | 7 | 12.3 | 7 | 6.1 | 142 | 10.8 | 145 | 5.5 |
| *15:02:01 | 0 | 0.0 | 0 | 0.0 | 13 | 22.8 | 14 | 12.3 | 290 | 22.0 | 306 | 11.6 |
| *16:02:01 | 0 | 0.0 | 0 | 0.0 | 1 | 1.8 | 1 | 0.9 | 19 | 1.4 | 20 | 0.8 |

Data are presented as number (%) of individuals carrying each allele (carrier-based analysis) or as allele counts and percentages. Percentages for carrier-based counts are calculated using the number of individuals as the denominator, whereas percentages for allele counts are calculated using the total number of alleles (2N). All alleles included in the analysis are presented. Some alleles observed in the General Control dataset were not included because they were not part of the predefined set of alleles analyzed in this study.

**Supplementary Table 2b. Carrier-based counts and allele counts of HLA-DRB3/4/5 alleles in each group**

|  | ***ICI-T1D (n=13)*** | | | | ***ICI-ILD (n=57)*** | | | | ***General Control (n=1320)*** | | | |
| --- | --- | --- | --- | --- | --- | --- | --- | --- | --- | --- | --- | --- |
|  | ***Carrier-based counts*** | | ***Allele counts*** | | ***Carrier-based counts*** | | ***Allele counts*** | | ***Carrier-based counts*** | | ***Allele counts*** | |
| ***DRB3/4/5*** | ***n*** | ***%*** | ***n*** | ***%*** | ***n*** | ***%*** | ***n*** | ***%*** | ***n*** | ***%*** | ***n*** | ***%*** |
| DRB3*01:01:02 | 0 | 0.0 | 0 | 0.0 | 3 | 5.3 | 3 | 2.6 | 176 | 13.3 | 180 | 6.8 |
| DRB3*02:02:01 | 3 | 23.1 | 3 | 11.5 | 11 | 19.3 | 13 | 11.4 | 207 | 15.7 | 217 | 8.2 |
| DRB3*03:01:01 | 2 | 15.4 | 2 | 7.7 | 6 | 10.5 | 7 | 6.1 | 143 | 10.8 | 148 | 5.6 |
| DRB3*03:01:03 | 0 | 0.0 | 0 | 0.0 | 2 | 3.5 | 2 | 1.8 | 46 | 3.5 | 48 | 1.8 |
| DRB4*01:02 | 0 | 0.0 | 0 | 0.0 | 0 | 0.0 | 0 | 0.0 | 24 | 1.8 | 24 | 0.9 |
| DRB4*01:03:01 | 10 | 76.9 | 13 | 50.0 | 24 | 42.1 | 29 | 25.4 | 473 | 35.8 | 535 | 20.3 |
| DRB4*01:03:02 | 2 | 15.4 | 2 | 7.7 | 12 | 21.1 | 12 | 10.5 | 428 | 32.4 | 467 | 17.7 |
| DRB5*01:01:01 | 0 | 0.0 | 0 | 0.0 | 7 | 12.3 | 7 | 6.1 | 143 | 10.8 | 148 | 5.6 |
| DRB5*01:02:01 | 0 | 0.0 | 0 | 0.0 | 13 | 22.8 | 14 | 12.3 | 287 | 21.7 | 304 | 11.5 |
| DRB5*02:02:01 | 0 | 0.0 | 0 | 0.0 | 1 | 1.8 | 1 | 0.9 | 19 | 1.4 | 20 | 0.8 |
| null | 6 | 46.2 | 6 | 23.1 | 23 | 40.4 | 26 | 22.8 | 469 | 35.5 | 533 | 20.2 |

Data are presented as number (%) of individuals carrying each allele (carrier-based analysis) or as allele counts and percentages. Percentages for carrier-based counts are calculated using the number of individuals as the denominator, whereas percentages for allele counts are calculated using the total number of alleles (2N). All alleles included in the analysis are presented. Some alleles observed in the General Control dataset were not included because they were not part of the predefined set of alleles analyzed in this study.

**Supplementary Table 3a. HLA-DRB1 allele frequencies in ICI-T1D and ICI-Control**.

|  | ***ICI-T1D (n=13)*** | | ***ICI Control (n=72)*** | | ***ICI-T1D vs. ICI Control*** | | |
| --- | --- | --- | --- | --- | --- | --- | --- |
| ***DRB1*** | ***n*** | ***%*** | ***n*** | ***%*** | ***P*** | ***OR*** | ***95%CI*** |
| *01:01:01 | 0 | 0.0 | 5 | 6.9 | >0.9999 | 0.00 | 0.000 to 4.060 |
| *04:01:01 | 0 | 0.0 | 1 | 1.4 | >0.9999 | 0.00 | 0.000 to 49.85 |
| *04:03:01 | 1 | 7.7 | 2 | 2.8 | 0.396 | 2.92 | 0.1869 to 26.15 |
| *04:05:01 | 7 | 53.8 | 26 | 36.1 | 0.354 | 2.06 | 0.6541 to 7.303 |
| *04:06:01 | 1 | 7.7 | 2 | 2.8 | 0.396 | 2.92 | 0.1869 to 26.15 |
| *04:10:01 | 0 | 0.0 | 1 | 1.4 | >0.9999 | 0.00 | 0.000 to 49.85 |
| *07:01:01 | 0 | 0.0 | 1 | 1.4 | >0.9999 | 0.00 | 0.000 to 49.85 |
| *08:02:01 | 4 | 30.8 | 6 | 8.3 | **0.042** | 4.89 | 1.318 to 18.51 |
| *08:03:02 | 2 | 15.4 | 13 | 18.1 | >0.9999 | 0.83 | 0.1667 to 3.780 |
| *09:01:02 | 4 | 30.8 | 16 | 22.2 | 0.494 | 1.56 | 0.4770 to 5.174 |
| *10:01:01 | 0 | 0.0 | 1 | 1.4 | >0.9999 | 0.00 | 0.000 to 49.85 |
| *11:01:01 | 1 | 7.7 | 3 | 4.2 | 0.492 | 1.92 | 0.1371 to 13.68 |
| *12:01:01 | 0 | 0.0 | 2 | 2.8 | >0.9999 | 0.00 | 0.000 to 12.19 |
| *12:02:01 | 0 | 0.0 | 6 | 8.3 | 0.584 | 0.00 | 0.000 to 3.041 |
| *13:02:01 | 2 | 15.4 | 8 | 11.1 | 0.646 | 1.45 | 0.2796 to 6.634 |
| *14:03:01 | 0 | 0.0 | 2 | 2.8 | >0.9999 | 0.00 | 0.000 to 12.19 |
| *14:05:01 | 0 | 0.0 | 3 | 4.2 | >0.9999 | 0.00 | 0.000 to 6.535 |
| *14:54:01 | 2 | 15.4 | 5 | 6.9 | 0.290 | 2.44 | 0.4358 to 14.48 |
| *15:01:01 | 0 | 0.0 | 13 | 18.1 | 0.203 | 0.00 | 0.000 to 1.357 |
| *15:02:01 | 0 | 0.0 | 23 | 31.9 | **0.016** | 0.00 | 0.000 to 0.5925 |

Data are presented as number of individuals carrying each allele (carrier-based analysis) or number (%). Allele counts are based on the presence of the allele irrespective of zygosity. Comparisons between groups were performed using Fisher’s exact test. P values < 0.05 are shown in bold.

**Supplementary Table 3b. HLA-DRB3/4/5 allele frequencies in ICI-T1D and ICI- Control**.

|  | ***ICI-T1D (n = 13)*** | | ***ICI Control (n = 72)*** | | ***ICI-T1D vs. ICI Control*** | | |
| --- | --- | --- | --- | --- | --- | --- | --- |
| ***DRB3/4/5*** | ***n*** | ***%*** | ***n*** | ***%*** | ***P*** | ***OR*** | ***95%CI*** |
| DRB3*01:01:02 | 0 | 0.0 | 4 | 5.6 | >0.9999 | 0.00 | 0.000 to 6.159 |
| DRB3*02:02:01 | 3 | 23.1 | 9 | 12.5 | 0.384 | 2.10 | 0.5340 to 9.108 |
| DRB3*03:01:01 | 2 | 15.4 | 9 | 12.5 | 0.673 | 1.27 | 0.2481 to 6.650 |
| DRB3*03:01:03 | 0 | 0.0 | 6 | 8.3 | 0.584 | 0.00 | 0.000 to 3.041 |
| DRB4*01:02 | 0 | 0.0 | 1 | 1.4 | >0.9999 | 0.00 | 0.000 to 49.85 |
| DRB4*01:03:01 | 10 | 76.9 | 32 | 44.4 | **0.038** | 4.17 | 1.028 to 14.82 |
| DRB4*01:03:02 | 2 | 15.4 | 16 | 22.2 | 0.726 | 0.64 | 0.1305 to 2.784 |
| DRB5*01:01:01 | 0 | 0.0 | 13 | 18.1 | 0.203 | 0.00 | 0.000 to 1.357 |
| DRB5*01:02:01 | 0 | 0.0 | 23 | 31.9 | **0.016** | 0.00 | 0.000 to 0.5925 |
| null | 6 | 46.2 | 24 | 33.3 | 0.529 | 1.71 | 0.4788 to 5.419 |

Data are presented as number of individuals carrying each allele (carrier-based analysis) or number (%). Allele counts are based on the presence of the allele irrespective of zygosity. Comparisons between groups were performed using Fisher’s exact test. P values < 0.05 are shown in bold.

**Supplementary Table 4. P values for HLA-DRB1–DRB3/4/5 haplotype frequency comparisons between ICI-ILD and general controls, with P values for each allele frequency by row (DRB1) and column (DRB3/4/5).**

| **Allele** | **DRB3** | | | | **DRB4** | | | **DRB5** | | |  |  |
| --- | --- | --- | --- | --- | --- | --- | --- | --- | --- | --- | --- | --- |
| **DRB1** | *01:01:02 | *02:02:01 | *03:01:01 | *03:01:03 | *01:02 | *01:03:01 | *01:03:02 | *01:01:01 | *01:02:01 | *02:02:01 | null | ***P* value** |
| *01:01:01 |  |  |  |  |  |  |  |  |  |  | 0.511 | 0.511 |
| *04:01:01 |  |  |  |  | >0.9999 |  |  |  |  |  |  | >0.9999 |
| *04:03:01 |  |  |  |  |  | 0.765 |  |  |  |  |  | 0.581 |
| *04:05:01 |  |  |  |  |  | 0.741 | 0.400 |  |  |  |  | >0.9999 |
| *04:06:01 |  |  |  |  |  | >0.9999 | 0.471 |  |  |  |  | 0.758 |
| *04:10:01 |  |  |  |  |  | 0.105 |  |  |  |  |  | 0.283 |
| *04:10:03 |  |  |  |  |  | **0.016** |  |  |  |  |  | 0.050 |
| *07:01:01 |  |  |  |  |  | 0.191 |  |  |  |  |  | 0.288 |
| *08:02:01 |  |  |  |  |  |  |  |  |  |  | 0.474 | 0.474 |
| *08:03:02 |  |  |  |  |  |  |  |  |  |  | **0.017** | **0.018** |
| *09:01:02 |  |  |  |  |  | 0.730 | 0.282 |  |  |  |  | 0.379 |
| *10:01:01 |  |  |  |  |  |  |  |  |  |  | >0.9999 | >0.9999 |
| *11:01:01 |  | >0.9999 |  |  |  |  |  |  |  |  |  | >0.9999 |
| *12:01:01 | 0.253 |  |  |  |  |  |  |  |  |  |  | 0.179 |
| *12:02:01 |  |  | >0.9999 | 0.716 |  |  |  |  |  |  |  | >0.9999 |
| *13:01:01 | 0.726 |  |  |  |  |  |  |  |  |  |  | 0.726 |
| *13:02:01 |  |  | >0.9999 | >0.9999 |  |  |  |  |  |  |  | >0.9999 |
| *14:03:01 | >0.9999 |  |  |  |  |  |  |  |  |  |  | >0.9999 |
| *14:05:01 |  | 0.250 |  |  |  |  |  |  |  |  |  | 0.429 |
| *14:06:01 |  | 0.674 |  |  |  |  |  |  |  |  |  | 0.674 |
| *14:54:01 |  | 0.103 |  |  |  |  |  |  |  |  |  | 0.180 |
| *15:01:01 |  |  |  |  |  |  |  | 0.664 |  |  |  | 0.664 |
| *15:02:01 |  |  |  |  |  |  |  |  | 0.870 |  |  | 0.871 |
| *16:02:01 |  |  |  |  |  |  |  |  |  | 0.573 |  | 0.573 |
| ***P* value** | 0.104 | 0.459 | >0.9999 | >0.9999 | 0.621 | 0.329 | 0.082 | 0.666 | 0.870 | 0.573 | 0.482 |  |

Each allele or haplotype frequency was analyzed using Fisher’s exact test. Rows represent HLA-DRB1 alleles and columns represent HLA-DRB3/4/5 alleles. Gray shading indicates haplotypes containing DRB4*01:03:01. P, *P* value. *P* values less than 0.05 are shown in bold. A total of 30 distinct HLA-DRB1–DRB3/4/5 haplotypes were identified in this study. One haplotype observed exclusively in the ICI-Control group was not included in this table because it was absent in the ICI-T1D, ICI-ILD, and General Control groups, resulting in a 2 × 2 contingency table with all zero counts, for which Fisher’s exact test is not informative.
